# The effect of phlebotomy training on blood sample rejection and phlebotomy knowledge of primary health care providers in Cape Town: A quasi-experimental study

**DOI:** 10.4102/phcfm.v9i1.1242

**Published:** 2017-04-13

**Authors:** Mumtaz Abbas, Fidele K. Mukinda, Mosedi Namane

**Affiliations:** 1 Department of Family Medicine and Public Health, University of Cape Town, South Africa; 2 Centre for Health Systems and Services Research and Development (CHSSRD), Department of Interdisciplinary Health Sciences, Stellenbosch University, South Africa; 3 Vanguard Community Heath Centre and Department of Family Medicine and Public Health, University of Cape Town, South Africa

## Abstract

**Background:**

There is an increasing amount of blood sample rejection at primary health care facilities (PHCFs), impacting negatively the staff, facility, patient and laboratory costs.

**Aim:**

The primary objective was to determine the rejection rate and reasons for blood sample rejection at four PHCFs before and after a phlebotomy training programme. The secondary objective was to determine whether phlebotomy training improved knowledge among primary health care providers (HCPs) and to develop a tool for blood sample acceptability.

**Study setting:**

Two community health centres (CHCs) and two community day centres (CDCs) in Cape Town.

**Methods:**

A quasi-experimental study design (before and after a phlebotomy training programme).

**Results:**

The sample rejection rate was 0.79% (*n* = 60) at CHC A, 1.13% (*n* = 45) at CHC B, 1.64% (*n* = 38) at CDC C and 1.36% (*n* = 8) at CDC D pre-training. The rejection rate remained approximately the same post-training (*p* > 0.05). The same phlebotomy questionnaire was administered pre- and post-training to HCPs. The average score increased from 63% (95% CI 6.97‒17.03) to 96% (95% CI 16.91‒20.09) at CHC A (*p =* 0.039), 58% (95% CI 9.09‒14.91) to 93% (95% CI 17.64‒18.76) at CHC B (*p* = 0.006), 60% (95% CI 8.84‒13.13) to 97% (95% CI 16.14‒19.29) at CDC C (*p =* 0.001) and 63% (95% CI 9.81‒13.33) to 97% (95% CI 18.08‒19.07) at CDC D (*p* = 0.001).

**Conclusion:**

There is no statistically significant improvement in the rejection rate of blood samples (*p* > 0.05) post-training despite knowledge improving in all HCPs (*p* < 0.05).

## Introduction

There is an increasing amount of blood sample rejection at primary health care facilities (PHCFs) which impacts negatively the staff, facility, patient and laboratory and results in high health care costs. Patient care and safety has become increasingly more important in laboratory medicine.^1^ Clinical laboratories have a big role to play in the management and diagnosis of patients.^2^ Clinical laboratories are striving to reduce the rejection rate of unsuitable blood samples and to provide an excellent level of care with more attention on patient care and safety.^3,4^

Approximately 70% – 80% of diagnoses are made in conjunction with laboratory tests. A delay in laboratory test results may result in delayed diagnoses, inappropriate or unnecessary treatment, increased risk to patient safety, high health care costs and waste of time.^5^ The National Health Laboratory Service (NHLS) provides laboratory services to all public sector hospitals in South Africa, and certain requirements are necessary before a sample can be successfully processed. There are three main phases to the testing of blood samples at the laboratory and these include the preanalytical, analytical and post-analytical phase. The preanalytical phase includes all steps from the time the clinician orders a test to the time the test is analysed; the analytical phase refers to the analysis of the sample; and the post-analytical phase refers to the reporting and interpretation of the test result.^6^

Studies reported the following preanalytical errors: patient identification (labelling errors, no test specified on request form, illegible request, no ward specified); sample collection (clotting, insufficient blood volume, incorrect sample tube, haemolysis); and sample transport (storage conditions i.e. incorrect temperature, sample lost or not received by the laboratory).^1,3^

Majority of blood samples are rejected as a result of preanalytical errors, and this accounts for up to 70% of laboratory errors.^7,8,9^ The prevalence of preanalytical problems ranged between 0.2% and 0.75% with the most common errors being haemolysis, clotting, insufficient blood volume, wrong sample tube and misidentification.^10^ Six to 12% of patients received inappropriate care because of laboratory errors, with up to 30% of errors resulting in patient discomfort, high hospital/health care costs and subjecting patients to re-sampling blood.^11^

A pilot audit at Vanguard Community Health Centre (CHC) in August 2012 (unpublished) showed that blood sample rejection is predominantly related to incorrect phlebotomy technique. This contributes significantly to preanalytical errors, as primary health care nurses do not receive formal phlebotomy training as part of their undergraduate training.

In a recent retrospective audit conducted at Tygerberg Hospital in Cape Town, 481 of 32 910 samples (1.46%) were rejected during the 2-week study period.^1^ The main reasons for sample rejection were clotting (30%) and inadequate sample volume (22%).^1^ Over half the samples were repeated and the average time for the sample to reach the laboratory was 5 days.

Haemolysis refers to the release of haemoglobin and intracellular components from erythrocytes into the surrounding plasma when the cell membrane is either damaged or disrupted.^12^ Haemolysis accounts for 40% – 70% of all unsuitable blood samples sent to the laboratory.^12^ Not all haemolysed samples are rejected by the laboratory, many samples can still be processed successfully except for electrolytes such as potassium and other tests that are influenced by haemolysis.^1^

The low rate of repeating blood samples of rejected samples and the delay in repeating the blood sample are also a concern as laboratory results can influence clinical decisions and inherent patient care.^13^ Turnaround times in repeating rejected samples can improve with access to the NHLS DISA Laboratory (DISALAB) on site where clinicians can check results during the current hospital visit.^1^

The evaluation of the reasons for blood sample rejection and its impact on health care costs as well as costs to the staff and most importantly to the patient is a form of assessing health systems performance. This provides role players in the health system with policy options and practical information that can be used to improve the health care system performance.^14^ One of the goals in strengthening health care systems in South Africa is to improve the functioning of clinical laboratories.^15^ This can be done by ensuring that good quality samples reach the laboratory and that staff are adequately trained by laboratory personnel on all aspects of phlebotomy that is required to ensure a sample of good quality.

The quality of a service can be determined by evaluating the success with which a service is delivered.^16^ Procedures that are deemed of high volume, high risk and expensive should be monitored by laboratories.^17^ In primary health care centres, phlebotomy is considered a high volume and high risk procedure in terms of the volume of patients that require blood drawn as well as the dangers of needle stick injuries to health care providers (HCPs) and the consequences of incorrect phlebotomy technique.

Phlebotomy can also be of high cost if blood samples are rejected because of various reasons. It was found that blood samples drawn by trained laboratory personnel/phlebotomists have lower blood sample rejection rates when compared to HCPs who have not received training (99.6% success vs 97.9%; *p* = 0.002). Even with trained personnel, haemolysis, clotting and insufficient blood volume were the main causes for blood sample rejection.^10^

Laboratory medicine plays a vital role in everyday clinical practice as well as in the long term follow-up of our patients. Only appropriate samples received by the laboratory can be analysed.^18^ Programmes that evaluated laboratory quality have shown blood sample rejection rates vary from 0.3% in outpatient departments to 0.8% in hospital inpatients.^10^

A study conducted at a government hospital in Bhavnagar showed that incorrect phlebotomy technique was the main reason for blood sample rejection. In order to improve the quality of samples that reach the laboratory, the authors of the above study suggest that phlebotomists and laboratory staff develop a manual on proper phlebotomy technique for HCPs.^18^

## Aims and objectives

The aim was to assess the effect of phlebotomy training on blood sample rejection rate and knowledge among primary HCPs at four facilities in Cape Town.

The primary objective of this study was to determine the rejection rate and reasons for blood sample rejection at four selected PHCFs pre- and post-phlebotomy training. The secondary objective was to determine whether phlebotomy training improved phlebotomy knowledge among primary HCPs and to develop a tool for blood sample acceptability.

## Study design and methods

### Study design

We performed a quasi-experimental study (before and after a phlebotomy training programme). We measured HCPs’ knowledge on phlebotomy technique and laboratory blood sample rejection rates before and after a phlebotomy training programme. All blood samples received and rejected by the NHLS during the month of May 2014 (pre-training) and July 2014 (post-training) were included in the study. A 2-hour Phlebotomy training programme was conducted at each facility during the month of June 2014. Phlebotomy knowledge pre- and post-phlebotomy training was assessed on the same day with each HCP completing the same phlebotomy questionnaire pre- and post-training.

Any sample processed by the laboratory that was not blood was excluded from the study.

### Intervention

The phlebotomy training programme was developed based on the ‘NHLS Western Cape Regional Laboratories Specimen Sampling Manual 2013’. The programme was developed using the information in the manual regarding health and safety measures of phlebotomy, proper venepuncture technique, the correct way to handle blood samples and phlebotomy equipment such as needles and syringes, the correct storage and transport of blood samples as well as the correct completion of the NHLS sample request form.

HCPs were assigned to receive phlebotomy training as our intervention of interest. The phlebotomy training session was approximately 2 hours long and was conducted at each of the four facilities (CHC A, CHC B, community day centre [CDC] C and CDC D) at a pre-determined day and time during the month of June 2014. All HCPs who attended the training session received a copy of the ‘NHLS Western Cape Regional Laboratories Specimen Sampling Manual 2013’.

The same pre- and post-training questionnaires were completed by each HCP in attendance at the training to assess if their knowledge had improved post-training. There was no pass mark; however, pre- and post-training scores determined whether knowledge around phlebotomy increased.

### Setting

The study was a multicentre study performed at four facilities in Cape Town, two 24-hour CHCs (CHC A and B) and two 8-hour CDCs (CDC C and D).

A community health facility (CHC and CDC) is a public PHCF funded by the government. It provides health care that covers a range of health promotion, disease prevention, curative and rehabilitative services to the community.^19,20^

The facilities included CHC A and B, which were large 24-hour facilities serving between 150 000 and 300 000 patients,^21,22^ and CDC C and D, which were smaller 8-hour facilities serving between 30 000 and 35 000 patients.^23^

### Study population and sampling strategy

The HCPs for the 2-hour phlebotomy training session were recruited by seeking their permission through the assistance of the facility managers and family physicians of the four facilities. There was a total of 23 HCPs who attended the training programme. Convenience sampling of HCPs was done. There were five HCPs from CHC A, seven from CHC B, seven from CDC C and four from CDC D. These HCPs were trained as trainers and were expected to train other HCPs at their respective facilities.

### Data collection

The data for May 2014 (pre-training) and July 2014 (post-training) were extracted from the NHLS DISALAB at the data warehouse in Johannesburg. The data were captured onto Microsoft^®^ Excel^®^. The variables extracted included the total number of routine blood samples performed, the number of blood samples rejected, the name of the test that was rejected (e.g. full blood count) and the reason for rejecting a blood sample (e.g. clotted sample).

Data from the data capture sheets were captured onto Microsoft Excel and was backed up on an external USB storage device, which was only accessible to the principal investigator and the research team. The data were encrypted and password protected within Microsoft Excel.

A questionnaire with 20 closed ended questions (see Appendix A) was developed based on the NHLS phlebotomy training manual 2013 and input from experts working in PHCFs. The questionnaire was piloted by staff ordering blood investigations or involved in phlebotomy at Vanguard CHC and amended where necessary. The questionnaire was either in English or Afrikaans.

### Data analysis

STATISTICA^TM^ version 14 was used for data analysis. The summary statistics was used to describe the variables. A test of proportion was performed to assess the effect of the intervention on the rejection rate of blood samples before and after training. The Wilcoxon signed-rank test and the paired *t*-test were used to analyse the pre- and post-training questionnaire data. A *p*-value of < 0.05 represented statistical significance.

## Results

Approximately 6–10 HCPs participated at these different sites. There were 23 study participants (Table 1) who attended the phlebotomy training session across all the facilities. The age, sex and level of phlebotomy experience of each HCP who attended the training were recorded. There were no exclusion criteria for the above variables of age, sex and level of experience. Most of the HCPs were aged between 30 and 50 years. There was only 1 male participant and 22 females. There were 3 student nurses, 17 nurses and only 3 doctors. The phlebotomy experience across participants varied, with nine participants having less than 5 years’ experience, five having 5–10 years’ experience and nine having more than 30 years’ experience.

**TABLE 1:** Demographic data of study participants across all four clinics.

Category	Participants *n* (%)
Rank	
Student nurse	3 (13)
Nurse	17 (74)
Doctor	3 (13)
Sex	
Male	1 (5)
Female	22 (95)
Age	
< 30 years	7 (30)
30–50 years	9 (39)
> 50 years	5 (22)
Unknown	2 (9)
Phlebotomy experience	
< 5 years	9 (39)
5–10 years	5 (22)
> 10 years	9 (39)

Box 1 describes the tool that was developed to facilitate the phlebotomy training, listing all the criteria for laboratory blood sample acceptability.

Box 1 ABBAS tool^©^.

1. **REQUEST FORM**
  - Name of facility
  - Diagnosis
  - Name of requesting doctor
  - Date and time of sample collection
  - HCP who took sample
  - Test specified on request form
  - Legible request
  - Patient’s details (first name & surname, folder no, age/DOB, sex)
  - Correct sample type for test requested
  - Doctor’s signature
  - INR sample – working contact number of patient or doctor
2. **SAMPLE**
  - Tube not expired (check expiry date on tube)/cracked
  - Correct colour tube
  - Labelled sample (sticker placed lengthwise, NOT covering cap):
    First name and surname, folder number OR sticker if available
  - Do not pre-label before drawing blood
  - Patient’s name on form and sample matching
  - Correct volume of blood (check volume required on tube)
  - Gentle mixing of sample 8–10 times
  - Sample tubes kept at room temperature, NO direct sunlight
  - Samples kept at room temperature, IN FRIDGE if after hours
3. **SAMPLE TRANSPORT**
  - Sealed packet
  - Blood sample/s and request form of index patient in one packet

ABBAS tool^©^ compiled by: Drs Abbas, Namane and Mukinda.

The total number of blood samples included in the study during May 2014 (pre-training): 7557 from CHC A, 3973 from CHC B, 2321 from CDC C and 589 from CDC D. The total number of blood samples included in the study during July 2014 (post-training): 10 218 from CHC A, 4114 from CHC B, 2279 from CDC C and 630 from CDC D.

During the month of May 2014 (Table 2): 60 of 7557 blood samples (0.79%) were rejected pre-training at CHC A and 79 of 10 218 blood samples (0.77%) post-training (*p* = 0.971). CHC A is the largest of the four facilities. Only 8 of 589 blood samples (1.36%) were rejected pre-training at CDC D and 7 of 630 blood samples (1.11%) post-training (*p* = 0.696). CDC D is the smallest of the four facilities.

**TABLE 2:** Rejection rate of blood samples before and after the phlebotomy training.

Before the phlebotomy training May 2014			After the phlebotomy training July 2014		
	
CHC/CDC	Blood registrations	Blood rejections *n* (%)	Blood registrations	Blood rejections *n* (%)	*p*
A	7557	60 (0.79)	10 218	79 (0.77)	0.876
B	3973	45 (1.13)	4114	48 (1.17)	0.886
C	2321	38 (1.64)	2279	37 (1.62)	0.971
D	589	8 (1.36)	630	7 (1.11)	0.696

The reasons for blood sample rejection were grouped into four main categories including technique related, knowledge related, request form related and unaccountable preanalytical errors. Technique-related errors include samples that were clotted or haemolysed. Knowledge related errors include errors because of incorrect blood volume or the incorrect blood sample tube used. Request form errors include any error in the completion of the request form such as no patient details indicated, illegible requests, name on sample tube and request form unmatched, no test requested or an unlabelled sample. Unaccountable preanalytical errors include all errors that are not accounted for by the above but that occur in the preanalytical phase of laboratory testing of blood samples. These include samples with missing reasons for rejection, samples older than 3 days, samples that leaked in transit as well as samples that were not received by the laboratory.

**FIGURE 1:**
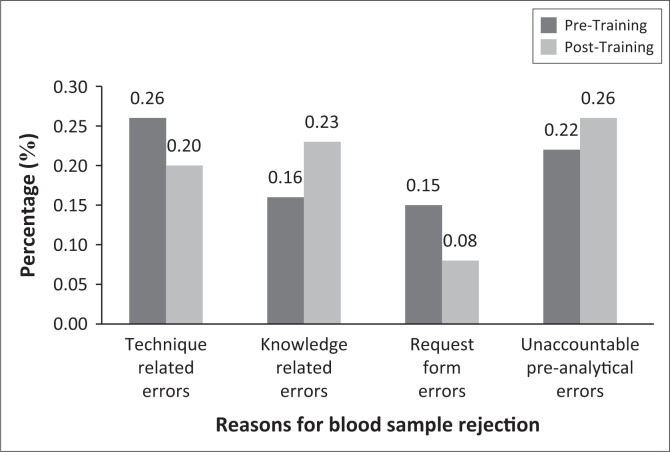
The rejection rate at CHC A.

Table 3 shows a detailed summary of rejection rates and related reasons by facility. At CHC A, many samples were rejected because of clotting, 20 (0.26%) were rejected pre-training and 16 (0.16%) post-training (Table 3). Request form errors improved post-training from 11 (0.15%) pre-training to 8 (0.08%) post-training. There was an increase in the rejection rate of samples because of unaccountable preanalytical errors, 17 (0.22%) pre-training to 27 (0.26%) post-training (Figure 1).

**TABLE 3:** Rejection rate and reasons for blood sample rejection before and after the phlebotomy training by facility.

Reasons for rejection	Before the phlebotomy training May 2014 *n* (%)				After the phlebotomy training July 2014 *n* (%)			
	
	CHC A	CHC B	CDC C	CDC D	CHC A	CHC B	CDC C	CDC D
Technique related	20 (0.26)	8 (0.20)	15 (0.65)	4 (0.68)	20 (0.20)	15 (0.36)	18 (0.79)	2 (0.32)
- Clotted	20 (0.26)	7 (0.18)	15 (0.65)	4 (0.68)	16 (0.16)	14 (0.34)	16 (0.70)	2 (0.32)
- Haemolysed	0	1 (0.01)	0	0	4 (0.04)	1 (0.02)	2 (0.09)	0
Knowledge related	12 (0.16)	16 (0.40)	5 (0.02)	0	24 (0.23)	9 (0.22)	3 (0.13)	3 (0.48)
- Incorrect blood volume	5 (0.07)	4 (0.10)	2 (0.09)	0	8 (0.08)	3 (0.07)	1 (0.04)	1 (0.16)
- Incorrect sample tube	7 (0.09)	12 (0.30)	3 (0.13)	0	16 (0.16)	6 (0.15)	2 (0.09)	2 (0.32)
Request form errors	11 (0.15)	11 (0.28)	3 (0.13)	0	8 (0.08)	12 (0.29)	6 (0.26)	0
- Name on form and sample unmatched	3 (0.04)	1 (0.03)	1 (0.04)	**-**	4 (0.04)	4 (0.10)	0	**-**
- No patient details	1 (0.01)	0	0	**-**	0	0	0	**-**
- No test requested	1 (0.01)	3 (0.08)	1 (0.04)	**-**	2 (0.02)	0	1 (0.04)	**-**
- Illegible request	1 (0.01)	0	0	**-**	0	0	0	**-**
- Unlabelled sample	5 (0.07)	3 (0.08)	0	**-**	2 (0.02)	5 (0.12)	3 (0.13)	**-**
- No reason stated	0	4 (0.10)	1 (0.04)	**-**	0	3 (0.07)	2 (0.09)	**-**
Unaccountable preanalytical errors	17 (0.22)	10 (0.25)	15 (0.65)	4(0.68)	27 (0.26)	12 (0.29)	10 (0.44)	2 (0.32)
- No reason stated	11 (0.15)	0	0	0	18 (0.18)	0	0	1 (0.16)
- Sample leaked	2 (0.03)	0	0	0	1 (0.01)	1 (0.02)	0	0
- No sample received	1(0.01)	2(0.05)	4(0.17)	4(0.68)	5 (0.05)	6 (0.15)	3 (0.13)	1 (0.16)
- Age of sample > 3 days	3 (0.03)	8 (0.20)	11 (0.04)	0	3 (0.03)	5 (0.12)	7 (0.31)	0

**FIGURE 2:**
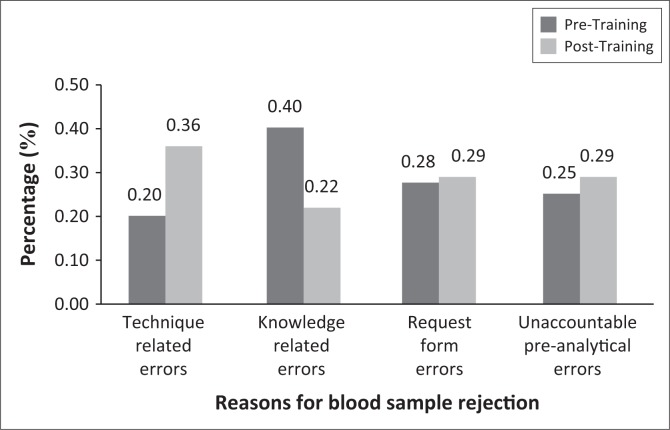
The rejection rate at CHC B.

At CHC B, knowledge related errors reduced from 16 (0.40%) rejected samples pre-training to 9 (0.22%). Twelve samples (0.16%) were rejected because of ‘incorrect sample tube’. This was reduced by half (0.15%) post-training. However, all other reasons for blood sample rejection increased post-training (Figure 2).

**FIGURE 3:**
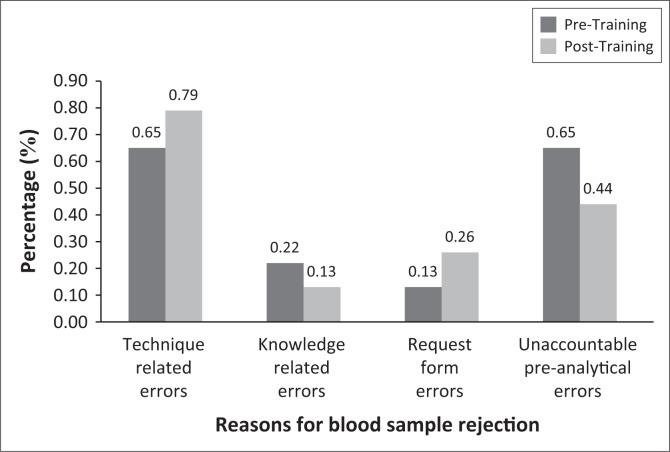
The rejection rate at CDC C.

At CDC C, many samples were rejected because of clotting pre- and post-training with the rejection rate increasing post-training. Knowledge related errors reduced from five (0.02%) rejected samples pre-training to three (0.13%). Request form errors doubled from three (0.13%) rejected samples pre-training to six (0.26%). Unaccountable preanalytical errors, because of ‘age of sample > 3 days’ also contributed to many of the blood sample rejections, 11 (0.04%) rejected samples pre-training and 7 (0.31%) post-training (Figure 3).

**FIGURE 4:**
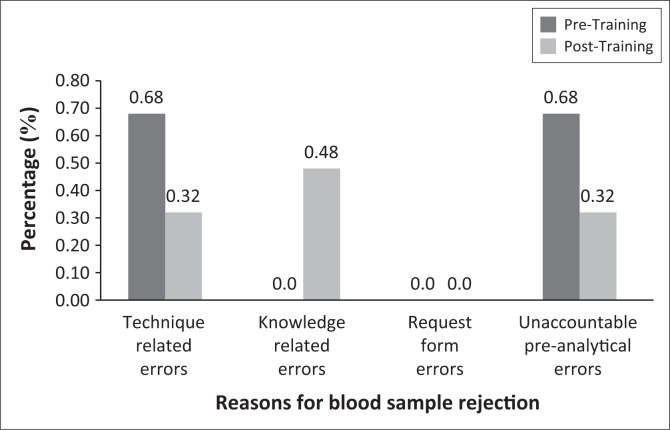
The rejection rate at CDC D.

CDC D is a smaller 8-hour facility compared to CDC C and only had 4 out of 589 (0.68%) samples rejected because of clotting pre-training, which was reduced to 2 (0.32%). Unaccountable preanalytical errors reduced by half post-training. There were no request form errors pre- or post-training (Figure 4).

The overall rejection rate, irrespective of facility, showed an increase in the rejection rate post-training, which was not statistically significant (Table 4).

**TABLE 4:** Overall rejection rates and related reasons for all facilities combined.

Before the phlebotomy training May 2014		After the phlebotomy training July 2014	*P*-value and 95% CI	
		
Related reason for rejection	Rejection rate *n* (%)	Rejection rate *n* (%)	*p*	95% CI
Technique related	47 (0.33)	55 (0.32)	0.38	-8 to 4
Knowledge related	33 (0.23)	39 (0.23)	0.74	-14.39 to 11.39
Request form errors	25 (0.17)	26 (0.15)	0.85	-4.23 to 3.73
Unaccountable preanalytical errors	46 (0.32)	51 (0.30)	0.73	-11.59 to 9.09

The pre- and post-training questionnaire scores showed an improvement in phlebotomy knowledge post-training as the average percentage score increased across all clinics. The average score increased from 63% (95% CI 6.97‒17.03) to 96% (95% CI 16.91‒20.09) at CHC A (*p* = 0.039), 58% (95% CI 9.09‒14.91) to 93% (95% CI 17.64‒18.76) at CHC B (*p* = 0.006), 60% (95% CI 8.84‒13.13) to 97% (95% CI 16.14‒19.29) at CDC C (*p* = 0.001) and 63% (95% CI 9.81‒13.33) to 97% (95% CI 18.08 – 19.07) at CDC D (*p =* 0.001).

**FIGURE 5:**
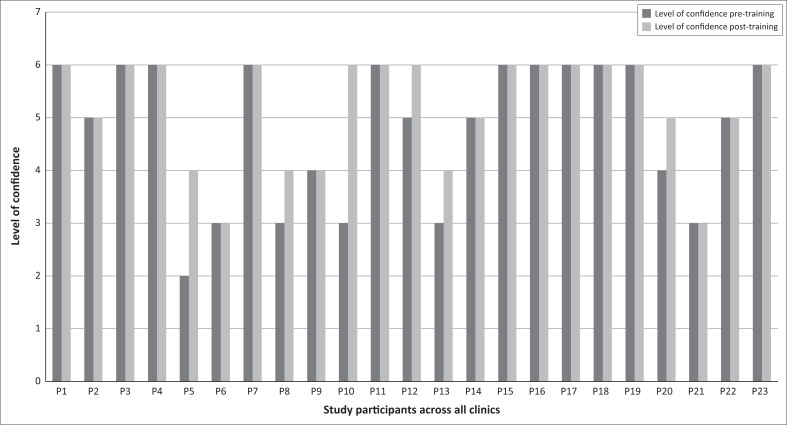
Level of confidence in phlebotomy across all clinics pre- and post-training using a Likert Scale.

Participants were asked to grade their level of confidence in phlebotomy on a Likert scale from 1 to 6, with 1 being ‘not confident at all’ and 6 being ‘very confident’. All participants’ level of confidence either remained the same or increased post-phlebotomy training (Figure 5).

## Ethical consideration

This study has been approved by the Health Research Ethics Committee (HREC) of the University of Cape Town (HREC REF: 549/2013) as well as by the Provincial Government of the Western Cape (REF: 2013RP190). The study was conducted according to the Declaration of Helsinki.

## Discussion

The study found that an appropriate intervention such as phlebotomy training has improved knowledge regarding phlebotomy (CHC A, *p* = 0.039; CHC B, *p* = 0.006; CDC C, *p* = 0.001; CDC D, *p* = 0.001). The study results show no statistically significant improvement in the rejection rate of blood samples sent to the laboratory by each of the four PHCFs (CHC A, *p* = 0.876; CHC B, *p* = 0.886; CDC C, *p* = 0.971; CDC D, *p* = 0.696) post-phlebotomy training. The overall rejection rate also showed no statistical significance in the rejection rate post-training (technique related errors, *p* = 0.38; knowledge related errors, *p* = 0.74; request form errors, *p* = 0.85; unaccountable preanalytical errors, *p* = 0.73).

The sample rejection rate was 0.79% at CHC A, 1.13% at CHC B, 1.64% at CDC C and 1.36% at CDC D pre-training. The rejection rates remained approximately the same post training. This could possibly be explained by other factors not explored in this quasi-experimental study. These factors include the phlebotomy experience of the primary HCPs, the same group of participants who received training were not followed up, and participants who did not receive training were involved in phlebotomy post-training which may have affected the study results.

A large number of blood samples in this study were rejected because of clotting, which was also seen in a similar study in 2011 at Tygerberg Hospital.^1^ Preanalytical errors is a major concern for laboratories accounting for up to 70% of laboratory errors,^4^ hence the need for a sustainable intervention to improve such errors. However, despite haemolysis being a leading cause of blood sample rejection accounting for up to 70% of unsuitable samples,^12^ this was not the leading cause of sample rejection in this study. From the overall rejection rates (Table 4), the leading cause of sample rejection was clotting which is related to phlebotomy technique, followed by unaccountable preanalytical errors. It is difficult to correct unaccountable preanalytical errors, as we do not always know why and how these errors occur.

Because approximately 60% of clinical decisions are influenced by laboratory results,^13^ an improvement in the rejection rate by improving preanalytical errors should improve patient care. Apart from the impact that rejected blood samples has on patient care in terms of the patient’s management, it also has a financial impact on the facility as well as causing pain and emotional harm to the patient.^24^

It was interesting that there was only 1 male participant out of the 23 participants. Only 3 out of 23 participants were doctors. This may be because of the high patient volumes and insufficient time to attend a 2-hour training programme, or they may not feel they will learn something new as phlebotomy training is part of the undergraduate training programme as a medical student. For each of the 23 participants, the pre- and post-training questionnaire scores improved. All participants’ level of confidence had improved or remained the same.

### Strengths and limitations

HCPs who did not participate in the training were involved in blood sample collection post-training, which may have adversely affected the study results. Reasons for some of the rejected blood samples were not provided in the NHLS database. There is also an underestimation of haemolysis as a cause of blood sample rejection as many haemolysed samples are still processed and only the potassium is rejected; therefore, the rejection rate might be higher than reported on in this study.

### Implications or recommendations

Increased knowledge in phlebotomy does not in itself influence practice. We recommend that primary HCPs receive phlebotomy training in order to reduce the rejection rate of laboratory blood samples and that any training at primary level facilities be accompanied by support from managers and that regular monitoring and evaluation of systems be done. The above recommendation is supported by similar audits conducted at other PHCFs.^25,26^

## Conclusion

The study results show no statistically significant improvement in the rejection rate of blood samples sent to the laboratory by each of the four PHCFs (*p* > 0.05) post-phlebotomy training despite an increase in the knowledge of phlebotomy in all HCPs (*p* < 0.05).

## Appendix 1

### PHLEBOTOMY QUESTIONAIRE 2013

#### DEMOGRAPHIC DATA:

Age:…………………………………………………………………

Occupation:………………………………………………………

Rank:………………………………………………………………

Years of experience in phlebotomy:………………………

Read each question carefully and choose the correct answer. Make an ‘x’ in the correct box.

1. It is important to check your phlebotomy equipment before proceeding with drawing blood.
  True
  False
2. Please circle the number that represents your confidence in drawing blood at your facility.
  Not confident at all    Very confident
     1 2 3 4 5 6
3. A blood sample, if refrigerated, should be stored at the following temperature to preserve the sample integrity.
  5–10°C
  2–8°C
  1–10°C
  37–38°C
4. Blood sample tubes should ideally be kept in the fridge.
  True
  False
5. CD4 blood samples once drawn should be kept in the fridge
  True
  False
6. The correct order of draw of blood samples is as follows:
  purple, yellow, blue, grey
  purple, blue, yellow, grey
  blue, purple, yellow, grey
  yellow, blue, grey, purple
7. Choose the correct option.
  The following areas should be avoided when drawing blood except:
  scars from burns and previous surgery
  veins from the antecubital fossa
  blood from a haematoma
  site where an intravenous line is placed
  oedematous extremities
8. If a superficial vein is not easily seen/felt, you can:
  Ask the patient to elevate their arm for a few seconds
  Wash the patient’s hand with cold water
  Tap the vein with one/two fingers and place a warm damp cloth onto the vein
  Gently massage the arm from the shoulder to the wrist
9. When drawing blood, the tourniquet should be placed:
  1–2cm from the puncture site
  5 cm from the puncture site
  3–4 cm from the puncture site
  6–8 cm from the puncture site
10. The tourniquet should not be left on the arm for more than:
  2 minutes
  10 minutes
  5 minutes
  30 seconds
11. The angle of insertion of the needle into the patient’s arm should be:
  45°
  15–30°
  90°
  10°20°
12. When should the tourniquet be released?
  while the first tube is filling
  1 min after the last tube is filling
  when the last tube is filling
  immediately after the last tube is filled
13. Choose the correct option:
  Blood samples should be mixed vigorously at least 8 times
  Blood samples should be mixed gently 8–10 times
  Blood samples should be inverted at least once
  Blood samples should be shaken to ensure adequate mixing of the sample
14. Factors that result in haemoconcentration include all of the following:
  prolonged tourniquet time, squeezing a puncture site, intravenous therapy
  True
  False
15. A haematoma can be prevented by:
  Applying pressure to the puncture site
  Forcefully drawing back the plunger of the syringe until the blood starts frothing
  Removing the needle before removing the tourniquet
16. Which of the following results in blood sample rejection?
  Haemolysis
  No facility name on request form
  Cracked sample tube
  Some of the above
  All of the above
17. Haemolysis can be prevented by the use of a Luer adaptor attached to a butterfly needle.
  True
  False
18. Samples should be transported in a sealed packet with one specimen per packet.
  True
  False
19. Blood samples should be kept out of direct sunlight.
  True
  False
20. Look at the blood sample and request form.
  Would this sample be accepted or rejected by the laboratory?
  Accepted
  Rejected
  [A completed request form and an expired sample tube will be provided, the correct answer to the question above will be: rejected as a result of an expired tube]
  The staff are expected to know that they need to check the expiry date on the tube.
